# The effects of short-term and long-term air pollution exposure on meibomian gland dysfunction

**DOI:** 10.1038/s41598-022-10527-y

**Published:** 2022-04-25

**Authors:** Ran Hao, Yu Wan, Liming Zhao, Yang Liu, Min Sun, Jing Dong, Yanhui Xu, Feng Wu, Jinwen Wei, Xiangyang Xin, Zhongping Luo, Shuxuan Lv, Xuemin Li

**Affiliations:** 1 Department of Ophthalmology, Peking University Third Hospital, No. 49, North Garden Street, Beijing, China; 2 Beijing Key Laboratory of Restoration of Damaged Ocular Nerve, Peking University Third Hospital, Beijing, China; 3 Department of Ophthalmology, Beijing Fengtai Hospital, Beijing, China; 4 Department of Ophthalmology, Daqing Oilfield General Hospital, Heilongjiang, China; 5 Department of Ophthalmology, Huabei Petroleum General Hospital, Hebei, China; 6 Department of Ophthalmology, The First Affiliated Hospital of Baotou Medical College, Inner Mongolia, China; 7 Department of Ophthalmology, Hebei Provincial Eye Hospital, Hebei, China; 8 Department of Ophthalmology, Fuyang Hospital Affiliated to Anhui Medical University, Anhui, China; 9 Department of Ophthalmology, Inner Mongolia Autonomous Region Xilingol League Hospital, Inner Mongolia, China; 10 Department of Ophthalmology, Inner Mongolia Baogang Hospital, Inner Mongolia, China; 11 Department of Ophthalmology, Tongliao City Ke’erqin Zuoyi Zhongqi People’s Hospital, Inner Mongolia, China; 12 Department of Ophthalmology, Yongqing People’s Hospital, Hebei, China

**Keywords:** Environmental social sciences, Eye diseases, Atmospheric science

## Abstract

We aim to assess the effects of different air pollutants on meibomian gland dysfunction (MGD). As a prospective multicenter study, 864 patients were recruited from four different regions (i.e., coal, oil, steel, and living). The oil region had a significantly lower temperature and higher O_3_ and SO_2_ concentrations than other regions. Notably, participants in oil region presented with more frequent and serious MGD signs and higher cytokine levels (median interleukin 6 [IL-6] in oil: 2.66, steel: 0.96, coal: 0.38, living: 0.56; IL-8 in oil: 117.52, steel: 46.94, coal: 26.89, living: 33; vascular endothelial growth factor [VEGF] in oil: 25.09, steel: 14.02, coal: 14.02, living: 28.47). The short-term fluctuations of cytokine levels were associated with the changes in gas levels (PM_2.5_ and IL-8: β = 0.016 [0.004–0.029]; O_3_ and IL-6: β = 0.576 [0.386–0.702]; O_3_ and IL-8: β = 0.479 [0.369–0.890]; SO_2_ and VEGF: β = 0.021 [0.001–0.047]). After long-term exposure, lid margin neovascularization (r = 0.402), meibomian gland (MG) expression (r = 0.377), MG secretion (r = 0.303), MG loss (r = 0.404), and tear meniscus height (r = − 0.345) were moderately correlated with air quality index (AQI). Individuals in oil region had more serious MGD signs and higher cytokine levels. MGD is susceptible to long-term exposure to high AQI.

## Introduction

Air pollution poses a serious global threat to public health^1–5^. According to World Health Organization (WHO), around 4.2 million deaths per year are due to ambient air pollution, and about 91% of the world’s population lives in areas where air quality levels are over WHO guidelines. Particulate matter (PM), ozone (O_3_), nitrogen dioxide (NO_2_), and sulfur dioxide (SO_2_) have been reported to endanger health and jointly constitute the dimensions of WHO air quality assessment.

Due to the rise in industrialization in the past decades, such as the growing steel casting, coal burning, oil extraction, and vehicle production, environmental pollution has increased in China. NO_2_ is produced by incomplete combustion inside motor engines, and traffic emission may be the primary source^1,2^. PM_2.5_ (PM with a diameter ≤ 2.5 µm) is part of PM_10_ (PM with a diameter ≤ 10 µm), and both of them originate from a variety of sources, including combustion, photochemical smog reactions, and sandstorms^1,2^. In China, people benefit from a high level of social development but suffer from the adverse health effects associated with air pollution. These pollutants are attributed to an extensive range of cardiovascular diseases^3–8^, pulmonary diseases^9^, metabolic diseases^10^, strokes^11^, and even sudden infant death syndrome^12^.

Increasing organ impairment has been associated with air pollution, and the ocular surface is not an exception since it is constantly exposed to the external environment. Symptoms of ocular surface pathologies tend to increase in a poor air environment, manifesting as irritation, foreign body sensation, redness, and blurred vision, among others^13^. Air pollutants have been shown to have adverse effects on dry eye disease (DED)^13–15^. Many studies have been conducted to investigate the changes in tear stability, tear volume, and corneal fluorescein staining (CFS) after exposure to air pollution. Chronic inflammation, allergy, and oxidative stress are involved in the pathogenesis of ocular surface diseases after air pollution exposure^13^. However, limited studies have assessed the changes in eyelid margin and meibomian gland dysfunction (MGD), which are supposed to exist before clinical manifestations are observed. The eyelid margin and MGD play key roles in the development of evaporative dry eye (DE). Additional, different regions may produce different pollutants and result in different ocular surface injury, which has not been mentioned in previous studies.

Because of the paucity of evidence regarding the effects of air pollutants on MGD in different locations in China, this study aimed to assess the changes in the ocular surface, particularly the lid margin, meibomian gland (MG) and tear cytokines, upon exposure to different air pollutants across different regions.

## Materials and methods

### Study design and participants

In this multicenter prospective cohort study, individuals were recruited from five provinces across China, including 11 hospitals in Beijing, Hebei, Heilongjiang, Anhui, and Inner Mongolia from February 2020 to February 2021, covering four different regions where factories mainly dedicated to steel, coal or oil production, and densely population (living) are predominant in the area. Participants aged 20–80 years who spend 3–4 h outdoor activities per day (average) in the corresponding zone and met diagnostic criteria of MGD^16,17^ were recruited in the study. Individuals with histories of another ocular surface abnormality, contact lens use, ocular surgeries, glaucoma medications use were excluded. Participants in each hospital were examined by the same trained doctors. Only first visit participants were included and divided into four groups according to their sampling regions (i.e., steel, coal, oil, and living). The study was performed in accordance with the Declaration of Helsinki and was approved by the Peking University Third Hospital Ethics Committee (Research Ethics Number M2019101). Informed consent was obtained from all participants.

### Outdoor air pollutants and meteorology data

During the observation period, the daily mean temperature, relative humidity, and wind speed were provided by the meteorological administrations of Beijing, Hebei, Heilongjiang, Anhui, and Inner Mongolia. The concentrations of daily air quality index (AQI), PM_2.5_, PM_10_, O_3_, NO_2_, and SO_2_ were obtained from open-access government air-quality monitoring data. Since the air pollutants exposure are constant, the 24-h average concentrations of PM_2.5_, PM_10_, NO_2_, and SO_2_ as well as the 8-h maximum value of O_3_ were collected as daily exposures according to previous studies^18–21^. AQI is determined by monitoring the five major air pollutants, i.e., PM, O_3_, NO_2_, SO_2_ and carbon monoxide (CO), and the maximum value among each of the pollutants is assigned as the date- and location-specific AQI value^20,21^. As Gope et al.^20^ and Mirabelli et al.^21^ reports, we recorded 24-h average AQI as daily AQI. According to different AQI levels (level I: 0–50; level II: 51–100; level III: 101–150), participants were divided into three groups. The daily exposure is considered as short-term exposure. And we recorded the 1-month average AQI (AQI mean) before the first observation as long-term exposure.

### Ocular surface health assessment

Symptoms were assessed using the ocular surface disease index (OSDI) questionnaire^22^. Lid margin morphology, MG morphology/function, tear meniscus height (TMH), Schirmer's test (ST), tear breakup time (TBUT), and CFS were examined in the individuals' right eyes, following previously reported methods^23–25^. Palpebral margin (hyperemia/telangiectasia, debris, edema/thickening, irregularity, and neovascularization) and MG (plugging, stenosis, expression secretion, and loss) were examined with the slit-lamp microscope. The MG loss was graded as follows^26^: 0 (no dropout), 1 (< 1/3 total area dropout), 2 (1/3–2/3 total area dropout), and 3 (> 2/3 total area dropout). The MG secretion was scored as follows^27^: 0 (clear meibum), 1 (cloudy meibum), 2 (granular meibum with debris), and 3 (inspissated meibum and toothpaste-like). MG expression was evaluated in five glands on the temporal, central, and nasal eyelid using the following standard: 0 (all glands expressible), 1 (three to four glands expressible), 2 (one to two glands expressible), and 3 (no glands expressible)^17^.

### Tear film collection and cytokine measurement

Tear samples were collected from the right eyes of participants. Without any anesthetic, non-irritating tear collection was conducted using 5-μL capillary pipettes. A plastic head was used to squeeze tears into 0.2 mL Eppendorf tubes, and tears were immediately frozen at − 80 °C. The cytokine levels in the tear samples were measured by a flow cytometer (BD FACS Canto ll, Becton Dickinson, Franklin Lakes, NJ, USA) and a bead array system (BD Cytometric Bead Array system, Becton Dickinson) following the instructions of the manufacturer. At least 50 μL undiluted tear samples were analyzed for cytokines including interleukin 1 beta (IL-1β), interleukin 6 (IL-6), interleukin 8 (IL-8), interleukin 10 (IL-10), interleukin 17 (IL-17), tumor necrosis factor-alpha (TNF-α), interferon-gamma (IFN-γ), vascular endothelial growth factor (VEGF), and B-cell activating factor (BAFF).

### Statistical analysis

Continuous variables were presented as the mean ± standard deviation (SD)/median (25% quantile, 75% quantile). Categorical variables were expressed as frequencies and percentages. Continuous variables were compared using analysis of variance or Kruskal–Wallis tests among four groups. Continuous data between two groups were compared using independent *t* tests or Mann–Whitney nonparametric *U* tests. Categorical variables were compared using the Chi-square test. Crosstab analyses were used to compare the status of the lid margin and MG morphology/function. Spearman correlation analysis was conducted to assess the associations between different atmospheric environments, various indices of ocular surface, and cytokine levels. Logistic regression analysis and multivariate linear regression analysis were used to evaluate the changes in ocular surface parameters and tear cytokines according to each air pollutant and meteorological variables. After variables collinearity checking and covariates (such as age and sex) being adjusted, regression models were developed. Since the MG function, conjunctivochalasis, OSDI, CFS and cytokine concentrations did not show a normal distribution, normality transition was performed before analysis. Statistical analysis was performed using SPSS version 23.0 (IBM Corp., Armonk, New York, USA). A *P* value < 0.05 was considered significant for all comparisons.

## Results

Between February 2020 and February 2021, 864 individuals were included, including 198 patients from steel region, 78 from coal region, 276 from living region, and 312 from oil region. The demographic characteristics in the four regions are shown in Table 1. The gender (*P* = 0.067) and age (*P* = 0.743) among the four regions showed no significant difference.

**Table 1:** Demographics characteristics, various air quality, ocular surface status and cytokine levels in different regions.

	Steel	Coal	Living	Oil	*P* value
Numbers	198	78	276	312	
**Gender**	1:2.09	1:2.25	1:1.97	1:2.39	0.067
Male	64	24	93	92	
Female	134	54	183	220	
Age (years)^$^	64.85 ± 6.70	65.82 ± 7.57	63.81 ± 9.93	67.64 ± 8.85	0.743
Temperature (°C)^&^	20 (− 5.5, 25)	17 (13, 22)	21 (1, 35)	− 3 (− 13, 28)	0.000*
Relative humidity (%)^&^	55 (35, 60)	81.5 (65, 92)	55 (43, 66)	68 (29, 80)	0.000*
Wind speed (level)^&^	3 (2, 3)	1 (0, 3)	2 (1, 3)	3.5 (0, 4)	0.000*
AQI^&^	66 (44, 71)	78 (60, 90)	54 (33, 69)	60 (50, 84)	0.000*
PM_2.5_ (μg/m^3^)^&^	23 (12.75, 43)	107 (7, 113)	45 (11.25, 95.75)	43 (23, 55)	0.000*
PM_10_ (μg/m^3^)^&^	45 (32, 72)	160 (18, 177)	64 (27, 85)	61 (50.25, 104)	0.000*
O_3_ (μg/m^3^)^&^	38 (12, 90)	17 (7, 71)	43.5 (11, 56)	66 (39, 79)	0.000*
NO_2_ (μg/m^3^)^&^	26 (16, 44)	36 (26, 54)	36 (17, 50)	28 (21, 45)	0.000*
SO_2_ (μg/m^3^)^&^	9 (6, 15)	15 (12, 19)	9 (5, 12)	16 (12, 23)	0.000*
**Lid margin**					
Hyperemia^#^	76 (42.7%)	27 (34.6%)	74 (26.8%)	115 (36.7%)	0.028*
Debris^#^	9 (4.5%)	21 (27.6%)	22 (8.0%)	43 (13.8%)	0.000*
Edema/thickening^#^	76 (38.4%)	9 (11.8%)	34 (12.3%)	40 (12.8%)	0.000*
Irregularity^#^	30 (15.2%)	3 (3.8%)	33 (12.0%)	27 (8.7%)	0.026*
Neovascularization^#^	15 (7.6%)	0 (0.0%)	3 (1.1%)	103 (33.0%)	0.000*
**Meibomian gland**					
Plugging^#^	57 (28.8%)	28 (35.9%)	165 (59.8%)	279 (89.4%)	0.000*
Stenosis^#^	82 (41.4%)	44 (56.4%)	64 (23.2%)	20 (6.4%)	0.000*
**Meibomian gland expression**^**#**^					
0	92 (46.5%)	6 (7.7%)	53 (19.2%)	2 (0.6%)	0.000*
1	44 (22.2%)	39 (50.0%)	134 (48.6%)	42 (13.5%)	
2	58 (29.3%)	33 (42.3%)	84 (30.4%)	184 (59.0%)	
3	4 (2.0%)	0 (0.0%)	5 (1.8%)	84 (26.9%)	
**Meibomian gland secretion**^**#**^					
0	79 (40.3%)	9 (11.5%)	87 (31.5%)	1 (0.3%)	0.000*
1	45 (23.0%)	52 (66.7%)	90 (32.6%)	68 (21.8%)	
2	43 (21.9%)	16 (20.5%)	52 (18.9%)	129 (41.4%)	
3	29 (14.8%)	1 (1.3%)	47 (17.0%)	114 (36.5%)	
**Meibomian gland loss**^**#**^					
0	142 (71.7%)	36 (46.2%)	149 (54.0%)	1 (0.3%)	0.000*
1	41 (20.7%)	33 (42.3%)	102 (37.0%)	113 (36.2%)	
2	11 (5.6%)	9 (11.5%)	23 (8.3%)	110 (35.3%)	
3	4 (2.0%)	0 (0.0%)	2 (0.7%)	88 (28.2%)	
**Conjunctivochalasis**^**#**^					
0	81 (41.3%)	28 (35.9%)	136 (49.3%)	93 (29.8%)	0.000*
1	72 (36.7%)	41 (52.6%)	135 (48.9%)	163 (52.2%)	
2	39 (19.9%)	9 (11.5%)	5 (1.8%)	38 (12.2%)	
3	4 (2.1%)	0 (0.0%)	0 (0.0%)	18 (5.8%)	
OSDI (score)^$^	22.55 (13.60, 32.39)	20.45 (14.77, 31.80)	15.91 (6.82, 22.15)	19.32 (14.77, 28.15)	0.000*
TMH (mm)^$^	0.25 ± 0.11	0.43 ± 0.16	0.28 ± 0.14	0.16 ± 0.06	0.000*
ST (mm)^$^	6.98 ± 3.25	8.70 ± 3.81	7.85 ± 3.35	6.49 ± 3.79	0.000*
TBUT (s)^$^	8.12 ± 4.51	7.85 ± 3.36	4.86 ± 2.17	4.10 ± 1.95	0.000*
CFS (score)^&^	1 (0, 2.5)	0 (0, 1)	0 (0, 0.25)	0 (0, 2)	0.000*
IL-1β (pg/ml)^&^	0 (0, 1.25)	0 (0, 3.26)	0 (0, 0)	0 (0, 1.35)	0.198
IL-6 (pg/ml)^&^	0.96 (0, 3.5)	0.38 (0, 8.54)	0.56 (0, 3.27)	2.66 (0.36, 13.67)	0.012*
IL-8 (pg/ml)^&^	46.94 (20.39, 87.94)	26.89 (5.02, 115.82)	33 (15.57,75.16)	117.52 (24.17,247.38)	0.001*
VEGF (pg/ml)^&^	14.02 (5.25, 25.97)	14.02 (1.92, 77.93)	28.47 (11.43, 59.21)	25.09 (14.31, 46.25)	0.002*

*AQI* air quality index, *PM* particulate matter, *O*_*3*_ ozone, *NO*_*2*_ nitrogen dioxide, *SO*_*2*_ sulfur dioxide, *OSDI* ocular surface disease index, *TMH* tear meniscus height, *ST* Schirmer’s I test, *TBUT* tear film break-up time, *CFS* corneal fluorescein staining, *IL-1β* interleukin 1 beta, *IL-6* interleukin 6, *IL-8* interleukin 8, *VEGF* vascular endothelial growth factor.

^#^Crosstab analyses were compared and results were expressed as number (percentage); ^$^Kruskal–Wallis tests were compared among four groups, independent *t* tests between two groups and results were shown as mean ± standard deviation (SD); ^&^Kruskal–Wallis tests were compared among four groups, Mann–Whitney nonparametric *U* tests between two groups and results were shown as median (25% quantile, 75% quantile); Chi-square tests were used for comparing gender among four regions. *P < 0.05.

### Different regions presented varying air quality

**Figure 1:**
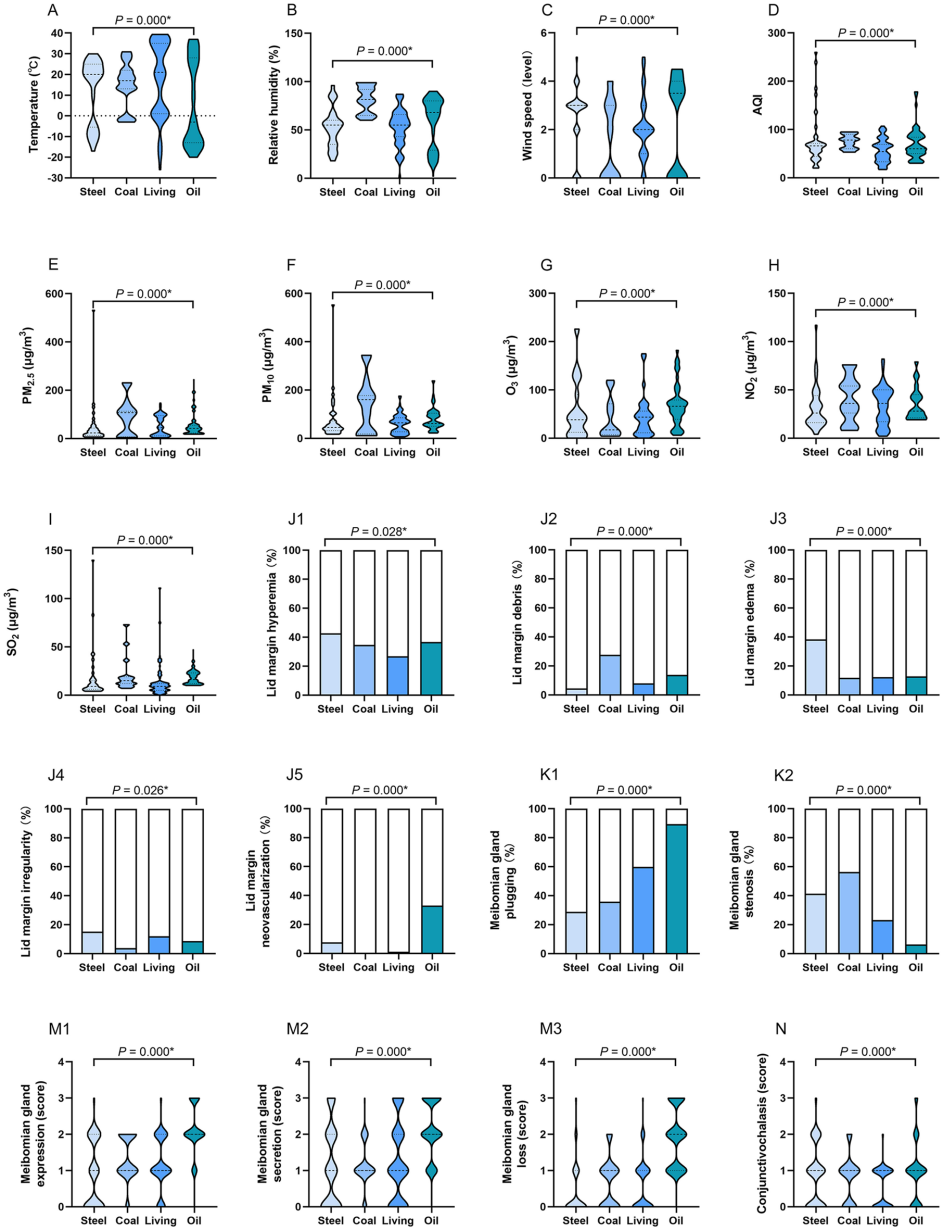

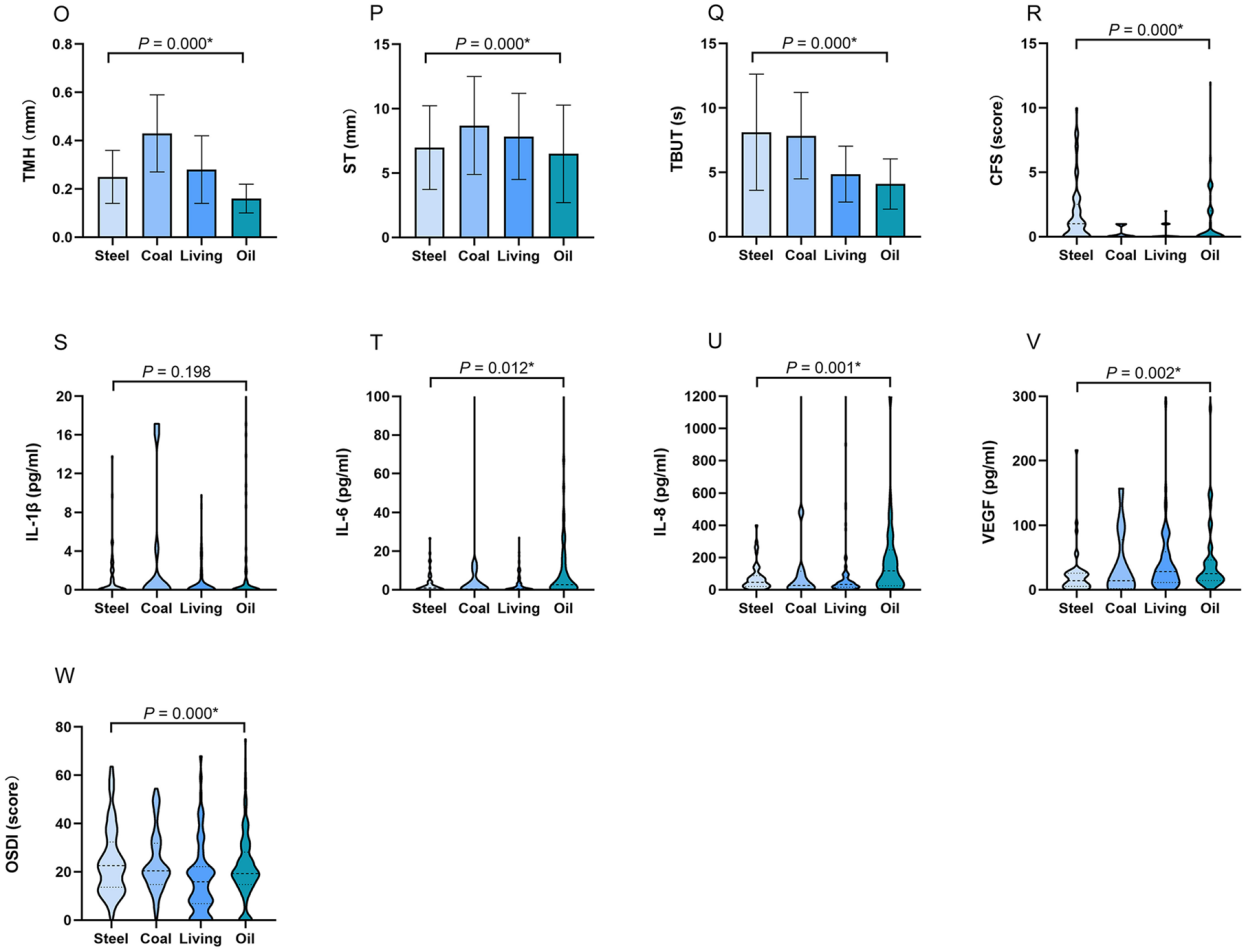
The differences of meteorological factors, ambient pollutants, ocular surface status and cytokine levels in four regions. In oil region, the temperature was significantly lower (**A**), the concentrations of O_3_ (**G**) and SO_2_ (**I**) were significantly higher than other regions. Relative humidity (**B**), AQI (**D**), PM_2.5_ (**E**), and PM_10_ (**F**) in coal region were significantly higher than other regions. The wind speed (**C**) in steel and oil region was significantly higher than coal and living regions. NO_2_ concentration (**H**) in steel region was significantly lower than other regions. Individuals in living region presented a low proportion of eyelid hyperemia (**J1**), coal and oil regions exhibited a high proportion of lid debris (**J2**). Participants in steel region had a relatively high proportion of lid edema (**J3**) and irregularity (**J4**). Individuals in oil and steel regions exhibited a high proportion of eyelid neovascularization (**J5**). Participants in oil region had a high incidence of MG plugging (**K1**), but few suffered from MG stenosis (**K2**). Individuals in oil region mainly exhibited higher levels of MG expression (**M1**), MG secretion (**M2**), MG loss (**M3**), and conjunctivochalasis (**N**) than other regions. Individuals in oil region demonstrated significantly lower TMH (**O**) and ST (**P**), shorter TBUT (**Q**), and higher CFS score (**R**), IL-6 (**T**), IL-8 (**U**), VEGF (**V**). Participants in living region reported less OSDI than other regions (**W**). The results were expressed as median with interquartile range in (**A**–**I**), (**M**,**N**) and (**R**–**W**); as percentage in (**J**–**K**); as mean ± standard deviation (SD) in (**O**–**Q**). *AQI* air quality index, *PM* particulate matter, *O*_*3*_ ozone, *NO*_*2*_ nitrogen dioxide, *SO*_*2*_ sulfur dioxide, *TMH* tear meniscus height, *ST* Schirmer’s I test, *TBUT* tear film break-up time, *CFS* corneal fluorescein staining, *IL-1β* interleukin 1 beta, *IL-6* interleukin 6, *IL-8* interleukin 8, *VEGF* vascular endothelial growth factor, *OSDI* ocular surface disease index, *P < 0.05.

The meteorological factors and ambient pollutants varied greatly and presented large disparity across the four regions, as shown in Table 1 and Fig. 1. The temperature (°C) in oil region was significantly lower than other regions (median temperature in oil: − 3, steel: 20, coal: 17, living: 21, *P* = 0.000), but the concentrations of O_3_ (μg/m^3^) and SO_2_ (μg/m^3^) were significantly higher than other regions (median O_3_ in oil: 66, steel: 38, coal: 17, living: 43.5; median SO_2_ in oil: 16, coal: 15, steel: 9, living: 9; all *P* = 0.000). Relative humidity (%), AQI, PM_2.5_ (μg/m^3^), and PM_10_ (μg/m^3^) in coal region were significantly higher than the other regions (median relative humidity: 81.5, AQI: 78, PM_2.5_: 107, PM_10_: 160, all *P* = 0.000). The wind speed (level) in steel and oil region was significantly higher than coal and living regions (median in steel: 3, oil: 3.5, coal: 1, living: 2, *P* = 0.000), but NO_2_ concentration (μg/m^3^) in steel region was significantly lower than the other regions (median in steel: 26, oil: 36, living: 36, coal: 28, *P* = 0.000).

### Individuals in four regions presented different ocular surface status and cytokine levels

The ocular surface status and cytokine levels of individuals according to the four regions are shown in Table 1 and Fig. 1. More than half of the participants demonstrated a variety of lid margin abnormalities. Individuals in coal and oil regions exhibited a high proportion of lid margin debris (coal: 27.6%, oil: 13.8%, living: 8.0%, steel: 4.5%, *P* = 0.000). Individuals in oil and steel regions exhibited a high proportion of eyelid neovascularization (oil: 33.0%, steel: 7.6%, living: 1.1%, coal: 0%, *P* = 0.000). Participants in steel region had a relatively high proportion of lid edema/thickening (38.4%, *P* = 0.000) and irregularity (15.2%, *P* = 0.026). Individuals in living region presented a low proportion of eyelid hyperemia (living: 26.8%, coal: 34.6%, oil: 36.7%, steel: 42.7%, *P* = 0.028).

The abnormalities of MG morphology in the four regions demonstrated a difference, particularly in oil region. Compared with other regions, participants in oil region had a high incidence of MG plugging (oil: 89.4%, living: 59.8%, coal: 35.9%, steel: 28.8%, *P* = 0.000), but few suffered from MG stenosis (oil: 6.4%, living: 23.2%, steel: 41.4%, coal: 56.4%, *P* = 0.000). Regarding MG expression, MG secretion, MG loss, and conjunctivochalasis, individuals in oil region mainly exhibited higher levels than other regions (all *P* = 0.000).

Similar with lid margin and MG evaluation, individuals in oil region demonstrated significantly lower TMH (oil: 0.16 ± 0.06, steel: 0.25 ± 0.11, coal: 0.43 ± 0.16, living: 0.28 ± 0.14, *P* = 0.000) and ST (oil: 6.49 ± 3.79; steel: 6.98 ± 3.25, coal: 8.70 ± 3.81, living: 7.85 ± 3.35, *P* = 0.000), shorter TBUT (oil: 4.10 ± 1.95, steel: 8.12 ± 4.51, coal: 7.85 ± 3.36, living: 4.86 ± 2.17, *P* = 0.000), and higher CFS score (oil: 0 [0, 2], steel: 1 [0, 2.5], coal: 0 [0, 1], living: 0 [0, 0.25], *P* = 0.000). In terms of OSDI, participants in living region reported less discomfort than other regions (median in living: 15.91, steel: 22.55, coal: 20.45, oil: 19.32, *P* = 0.000).

Regarding cytokines to be tested, IL-10, IL-17, INF-γ, and TNF-α were not detected. Individuals in oil and coal regions exhibited the highest levels of IL-1β (oil: 0 [0, 1.35], coal: 0 [0, 3.26], steel: 0 [0, 1.25], living: 0 [0, 0], *P* = 0.198). Additionally, participants in oil region demonstrated the highest levels of IL-6, IL-8 and VEGF (median IL-6 in oil: 2.66, steel: 0.96, coal: 0.38, living: 0.56, *P* = 0.012; IL-8 in oil: 117.52, steel: 46.94, coal: 26.89, living: 33, *P* = 0.001; VEGF in oil: 25.09, steel: 14.02, coal: 14.02, living: 28.47, *P* = 0.002).

### The diversity of ocular surface status in different regions was associated with air quality indices

The associations between air quality and MGD signs are shown in Table 2. In this logistic regression model, PM_2.5_, O_3_ and SO_2_ exposure showed an odd ratio (OR) 1.016 [95% confidence interval (CI) 1.004–1.038], OR 1.024 (95% CI 1.004–1.045), OR 1.037 (95% CI 1.015–1.059) for lid margin neovascularization, respectively (*P* = 0.043, *P* = 0.016, *P* = 0.001). PM_10_ exposure had an OR 1.017 (95% CI 1.002–1.031) for lid margin debris (*P* = 0.025). Temperature presented an OR 1.060 (95% CI 1.041–1.080) for lid margin hyperemia (*P* = 0.000), OR 1.037 (95% CI 1.007–1.068) for debris (*P* = 0.016), OR 1.040 (95% CI 1.009–1.073) for edema (*P* = 0.012), OR 0.934 (95% CI 0.904–0.965) for neovascularization (*P* = 0.000) and OR 0.965 (95% CI 0.948–0.982) for MG plugging (*P* = 0.000). Relative humidity had an OR 0.967 (95% CI 0.942–0.993) for lid margin irregularity (*P* = 0.012) and OR 0.977 (95% CI 0.963–0.992) for MG stenosis (*P* = 0.002). Age presented an OR 1.017 (95% CI 1.001–1.032) for lid margin hyperemia (*P* = 0.039), OR 1.097 (95% CI 1.051–1.146) for debris (*P* = 0.000), OR 1.107 (95% CI 1.045–1.171) for edema (*P* = 0.000), OR 1.094 (95% CI 1.044–1.147) for neovascularization (*P* = 0.000) and OR 1.016 (95% CI 1.003–1.029) for MG plugging (*P* = 0.015). No significant association of gender, wind speed, AQI and NO_2_ with MGD signs was observed.

**Table 2:** Logistic regression: effects of air quality on meibomian gland dysfunction signs.

	Age (per 1 year)	Temperature (per 1 °C)	Relative humidity (per 1%)	PM_2.5_ (per 1 μg/m^3^)	PM_10_ (per 1 μg/m^3^)	O_3_ (per 1 μg/m^3^)	NO_2_ (per 1 μg/m^3^)	SO_2_ (per 1 μg/m^3^)
**Lid margin**								
Hyperemia	1.017 (1.001–1.032)*	1.060 (1.041–1.080)**	0.989 (0.977–1.002)	0.997 (0.985–1.010)	1.002 (0.992–1.012)	0.997 (0.989–1.005)	0.987 (0.966–1.008)	1.000 (0.943–1.061)
Debris	1.097 (1.051–1.146)**	1.037 (1.007–1.068)*	0.982 (0.963–1.002)	0.999 (0.982–1.016)	1.017 (1.002–1.031)*	1.009 (0.997–1.020)	1.022 (0.993–1.051)	1.002 (0.958–1.049)
Edema/Thickening	1.107 (1.045–1.171)**	1.040 (1.009–1.073)*	1.005 (0.983–1.028)	0.993 (0.973–1.013)	1.016 (0.999–1.033)	0.987 (0.973–1.000)	0.940 (0.903–0.980)	0.961 (0.913–1.012)
Irregularity	0.969 (0.945–0.994)	0.978 (0.947–1.010)	0.967 (0.942–0.993)*	1.012 (0.990–1.035)	0.983 (0.963–1.003)	0.991 (0.972–1.010)	1.026 (0.992–1.061)	0.957 (0.901–1.017)
Neovascularization	1.094 (1.044–1.147)**	0.934 (0.904–0.965)**	1.020 (0.993–1.048)	1.016 (1.004–1.038)*	0.993 (0.974–1.012)	1.024 (1.004–1.045)*	0.919 (0.875–0.966)	1.037 (1.015–1.059)**
**Meibomian gland**								
Plugging	1.016 (1.003–1.029)*	0.965 (0.948–0.982)**	0.989 (0.977–1.002)	1.008 (0.996–1.020)	0.989 (0.980–0.998)	0.998 (0.991–1.006)	1.002 (0.983–1.021)	1.011 (0.991–1.033)
Stenosis	0.957 (0.903–0.970)	1.011 (0.993–1.030)	0.977 (0.963–0.992)**	0.993 (0.980–1.006)	1.006 (0.996–1.016)	0.996 (0.987–1.005)	1.010 (0.989–1.031)	0.993 (0.970–1.017)

*PM* particulate matter, *O*_*3*_ ozone, *NO*_*2*_ nitrogen dioxide, *SO*_*2*_ sulfur dioxide.

The odds ratio (95% confidence interval) is shown for all significant correlations in bold.

*P < 0.05, **P < 0.01.

The effects of air quality on ocular surface and tear cytokines are shown in Table 3. Multicollinearity between all air pollution variables was examined by ensuring that the variance inflation factors did not exceed 10. Significant associations were found between increased OSDI scores and higher PM_2.5_ [β = 0.146 (95% CI 0.094–0.198), *P* = 0.000, per 1 μg/m^3^ increase], higher PM_10_ [β = 0.118 (95% CI 0.080–0.156), *P* = 0.000, per 1 μg/m^3^ increase], higher O_3_ [β = 0.233 (95% CI 0.179–0.353), *P* = 0.020, per 1 μg/m^3^ increase], and higher SO_2_ [β = 0.137 (95% CI 0.093–0.210), *P* = 0.000, per 1 μg/m^3^ increase]. Significant associations were shown between decreased TMH and higher PM_2.5_ [β = − 0.074 (95% CI − 0.110 to − 0.012), *P* = 0.031] and higher O_3_ [β = − 0.075 (95% CI − 0.127 to − 0.010), *P* = 0.000]. Higher O_3_ exposure also associated with decreased ST [β = − 0.112 (95% CI − 0.145 to − 0.010), *P* = 0.043]. Decreased TBUT showed associations with higher PM_2.5_ [β = − 0.023 (95% CI − 0.032 to − 0.014), *P* = 0.000], higher O_3_ [β = − 0.024 (95% CI − 0.039 to − 0.010), *P* = 0.001] and higher SO_2_ [β = − 0.077 (95% CI − 0.092 to − 0.051), *P* = 0.000]. Significant associations were shown between increased CFS scores and higher PM_2.5_ [β = 0.021 (95% CI 0.013–0.028), *P* = 0.000], higher PM_10_ [β = 0.012 (95% CI 0.007–0.018), *P* = 0.000], higher O_3_ [β = 0.028 (95% CI 0.018–0.039), *P* = 0.001] and higher SO_2_ [β = 0.089 (95% CI 0.054–0.123), *P* = 0.000]. MG loss was associated with higher PM_2.5_ [β = 0.015 (95% CI 0.011–0.020), *P* = 0.000] and higher O_3_ [β = 0.015 (95% CI 0.010–0.020), *P* = 0.000]. However, no association was found between air pollutants and MG expression and secretion. PM was associated with tear cytokines, such as PM_2.5_ and IL-6 [β = 0.015 (95% CI 0.001–0.028), *P* = 0.035], IL-8 [β = 0.016 (95% CI 0.004–0.029), *P* = 0.013] and VEGF [β = 0.012 (95% CI 0.002–0.022), *P* = 0.044]; PM_10_ and IL-6 [β = 0.018 (95% CI 0.006–0.032), *P* = 0.006]. Higher O_3_ exposure was associated with increased IL-1β [β = 0.011 (95% CI 0.000–0.022), *P* = 0.045], IL-6 [β = 0.576 (95% CI 0.386–0.702), *P* = 0.007] and IL-8 [β = 0.479 (95% CI 0.369–0.890), *P* = 0.022], however, was not associated with VEGF concentrations. Increased VEGF was associated with higher NO_2_ [β = 0.012 (95% CI 0.003–0.022), *P* = 0.012] and higher SO_2_ [β = 0.021 (95% CI 0.001–0.047), *P* = 0.047]. Decreased temperature was associated with lower TBUT [β = 0.035 (95% CI 0.019–0.051), *P* = 0.001] and serious MG secretion [β = − 0.010 (95% CI − 0.016 to − 0.004), *P* = 0.000]. Because of collinearity, the AQI were not included in the analysis model.

**Table 3:** Multiple linear regression analysis: effects of air quality on ocular surface and tear cytokines.

	Age (per 1 year)	Temperature (per 1 °C)	Relative humidity (per 1%)	Wind speed (per 1 level)	PM_2.5_ (per 1 μg/m^3^)	PM_10_ (per 1 μg/m^3^)	O_3_ (per 1 μg/m^3^)	NO_2_ (per 1 μg/m^3^)	SO_2_ (per 1 μg/m^3^)
OSDI	0.003 (0.001 to 0.022)	0.049 (− 0.020 to 0.118)	**− 0.101 (− 0.153** to − 0.050)*	− 0.868 (− 1.724 to − 0.013)	0.146 (0.094 to 0.198)**	0.118 (0.080 to 0.156)**	0.233 (0.179 to 0.353)*	0.063 (− 0.019 to 0.145)	0.137 (0.093 to 0.210)**
TMH	− 0.002 (− 0.002 to − 0.001)	0.003 (0.002 to 0.004)	0.000 (0.000 to 0.001)	0.012 (0.002 to 0.022)	− 0.074 (− 0.110 to − 0.012)*	− 0.005 (− 0.008 to − 0.003)	− 0.075 (− 0.127 to − 0.010)**	− 0.001 (− 0.002 to − 0.000)	0.005 (0.004 to 0.006)
ST	− 0.018 (− 0.050 to 0.013)	0.009 (− 0.019 to 0.037)	− 0.002 (− 0.021 to 0.016)	− 0.171 (− 0.477 to 0.136)	0.018 (0.000 to 0.027)	− 0.002 (− 0.017 to 0.013)	− 0.112 (− 0.145 to − 0.010)*	0.022 (− 0.011 to 0.054)	− 0.012 (− 0.047 to 0.023)
TBUT	− 0.013 (− 0.026 to 0.000)*	0.035 (0.019 to 0.051)**	0.000 (− 0.012 to 0.011)	− 0.426 (− 0.620 to − 0.232)	− 0.023 (− 0.032 to − 0.014)**	− 0.010 (− 0.022 to 0.002)	− 0.024 (− 0.039 to − 0.010)**	− 0.012 (− 0.031 to 0.006)	− 0.077 (− 0.092 to − 0.051)**
CFS	0.025 (0.017 to 0.033)	− 0.014 (− 0.024 to − 0.004)	− 0.018 (− 0.026 to − 0.011)	0.071 (− 0.055 to 0.196)	0.021 (0.013 to 0.028)**	0.012 (0.007 to 0.018)**	0.028 (0.018 to 0.039)**	0.004 (− 0.008 to 0.016)	0.089 (0.054 to 0.123)**
MG expression	0.014 (0.010 to 0.018)**	− 0.006 (− 0.011 to − 0.001)	− 0.004 (− 0.007 to 0.000)	− 0.035 (− 0.097 to 0.028)	0.007 (0.003 to 0.011)	0.003 (0.000 to 0.005)	0.008 (0.002 to 0.014)	0.003 (− 0.008 to 0.013)	0.004 (0.000 to 0.011)
MG secretion	0.029 (0.025 to 0.034)**	− 0.010 (− 0.016 to − 0.004)**	− 0.005 (− 0.009 to − 0.001)	0.130 (0.059 to 0.201)	0.000 (− 0.006 to 0.004)	0.002 (− 0.002 to 0.005)	0.006 (0.000 to 0.011)	0.001 (− 0.007 to 0.006)	0.020 (0.003 to 0.038)
MG loss	0.015 (0.010 to 0.020)**	− 0.009 (− 0.015 to − 0.003)	0.000 (− 0.004 to 0.005)	**0.110 (0.036 to 0.185)**	0.015 (0.011 to 0.020)**	0.007 (0.004 to 0.011)	0.015 (0.010 to 0.020)**	0.008 (− 0.007 to 0.020)	− 0.003 (− 0.011 to 0.005)
Conjunctivochalasis	0.025 (0.022 to 0.028)**	− 0.003 (− 0.007 to 0.001)	− 0.006 (− 0.009 to − 0.003)	− 0.006 (− 0.009 to − 0.003)	− 0.002 (− 0.005 to 0.001)	0.002 (− 0.001 to 0.004)	0.000 (− 0.002 to 0.002)	0.003 (− 0.002 to 0.008)	0.002 (− 0.003 to 0.007)
IL-1β	0.032 (− 0.019 to 0.083)	− 0.004 (− 0.102 to 0.094)	0.037 (− 0.009 to 0.083)	− 0.424 (− 1.073 to 0.226)	0.007 (− 0.009 to 0.024)	− 0.001 (− 0.005 to 0.002)	0.011 (0.000 to 0.022)*	0.007 (− 0.001 to 0.015)	− 0.044 (− 0.112 to 0.023)
IL-6	− 0.459 (− 1.956 to 1.038)	− 1.697 (− 4.980 to 1.585)	− 0.310 (− 1.847 to 1.227)	− 4.708 (− 6.327 to 6.912)	0.015 (0.001 to 0.028)*	0.018 (0.006 to 0.032)**	0.576 (0.386 to 0.702)**	0.396 (− 0.003 to 2.285)	− 0.003 (− 0.020 to 0.014)
IL-8	0.014 (0.005 to 0.022)	0.001 (− 0.015 to 0.016)	− 0.001 (− 0.008 to 0.006)	− 0.091 (− 0.194 to 0.013)	0.016 (0.004 to 0.029)**	0.005 (0.001 to 0.010)	0.479 (0.369 to 0.890)*	0.008 (0.001 to 0.017)	0.005 (− 0.010 to 0.024)
VEGF	0.010 (0.002 to 0.019)	− 0.008 (− 0.024 to 0.008)	0.004 (− 0.004 to 0.011)	0.042 (− 0.065 to 0.148)	0.012 (0.002 to 0.022)*	0.007 (− 0.002 to 0.012)	0.003 (0.001 to 0.007)	0.012 (0.003 to 0.022)*	0.021 (0.001 to 0.047)*

*PM* particulate matter, *O*_*3*_ ozone, *SO*_*2*_ sulfur dioxide, *NO*_*2*_ nitrogen dioxide, *OSDI* ocular surface disease index, *ST* Schirmer’s I test, *TMH* tear meniscus height, *TBUT* tear film break-up time, *CFS* corneal fluorescein staining, *MG* meibomian gland, *IL-1β* interleukin 1 beta, *IL-6* interleukin 6, *IL-8* interleukin 8, *VEGF* vascular endothelial growth factor.

The β (95% confidence interval) is shown for all significant correlations in bold.

*P < 0.05, **P < 0.01.

The correlations between cytokine concentrations and ocular surface status during short-term exposure are shown in Table 4. Notably, the IL-6 concentration was moderately correlated with conjunctivochalasis (r = 0.312, *P* = 0.000), CFS (r = 0.307, *P* = 0.038), and TBUT (r = − 0.323, *P* = 0.000). The VEGF concentration was moderately correlated with CFS (r = 0.323, *P* = 0.028).

**Table 4:** Correlations between the cytokine concentrations and ocular surface status during short-term exposure.

	IL-1β	IL-6	IL-8	VEGF
**Lid margin hyperemia**				
r	0.128	0.194**	0.197**	0.046
*P*	0.069	0.005	0.005	0.512
**Lid margin debris**				
r	0.148*	0.274**	0.264**	0.237**
*P*	0.033	0.000	0.000	0.000
**Lid margin edema**				
r	0.047	0.079	0.081	− 0.013
*P*	0.496	0.259	0.243	0.847
**Lid margin irregularity**				
r	− 0.079	− 0.108	− 0.001	− 0.063
*P*	0.259	0.121	0.986	0.363
**Lid margin vascularization**				
r	0.061	− 0.034	− 0.042	− 0.055
*P*	0.378	0.623	0.548	0.429
**Meibomian gland plugging**				
r	0.055	0.005	0.048	0.024
*P*	0.431	0.945	0.487	0.730
**Meibomian gland stenosis**				
r	− 0.018	0.089	0.071	0.044
*P*	0.794	0.206	0.317	0.536
**Meibomian gland expression**				
r	0.208**	0.255**	0.294**	0.129
*P*	0.000	0.000	0.000	0.062
**Meibomian gland secretion**				
r	0.078	0.271**	0.282**	0.108
*P*	0.263	0.000	0.000	0.120
**Meibomian gland loss**				
r	0.079	0.203**	0.266**	0.059
*P*	0.270	0.000	0.000	0.410
**Conjunctivochalasis**				
r	0.151**	0.312**	0.288**	0.053
*P*	0.000	0.000	0.000	0.445
**OSDI**				
r	0.283*	0.045	0.014	− 0.010
*P*	0.028	0.518	0.838	0.891
**TMH**				
r	0.090	− 0.002	0.055	0.063
*P*	0.196	0.978	0.428	0.365
**ST**				
r	0.056	− 0.185*	− 0.126	− 0.057
*P*	0.477	0.018	0.107	0.470
**TBUT**				
r	0.013	− 0.323*	− 0.039	− 0.076
*P*	0.850	0.038	0.580	0.273
**CFS**				
r	0.099	0.307**	0.245**	0.323*
*P*	0.155	0.000	0.000	0.028

*OSDI* ocular surface disease index, *TMH* tear meniscus height, *ST* Schirmer’s I test, *TBUT* tear film break-up time, *CFS* corneal fluorescein staining, *IL-1β* interleukin 1 beta, *IL-6* interleukin 6, *IL-8* interleukin 8, *VEGF* vascular endothelial growth factor.

*P < 0.05, two-tail, **P < 0.01, two-tail for Spearman correlation analysis.

### Air quality index monthly average was related to MGD

**Figure 2:**
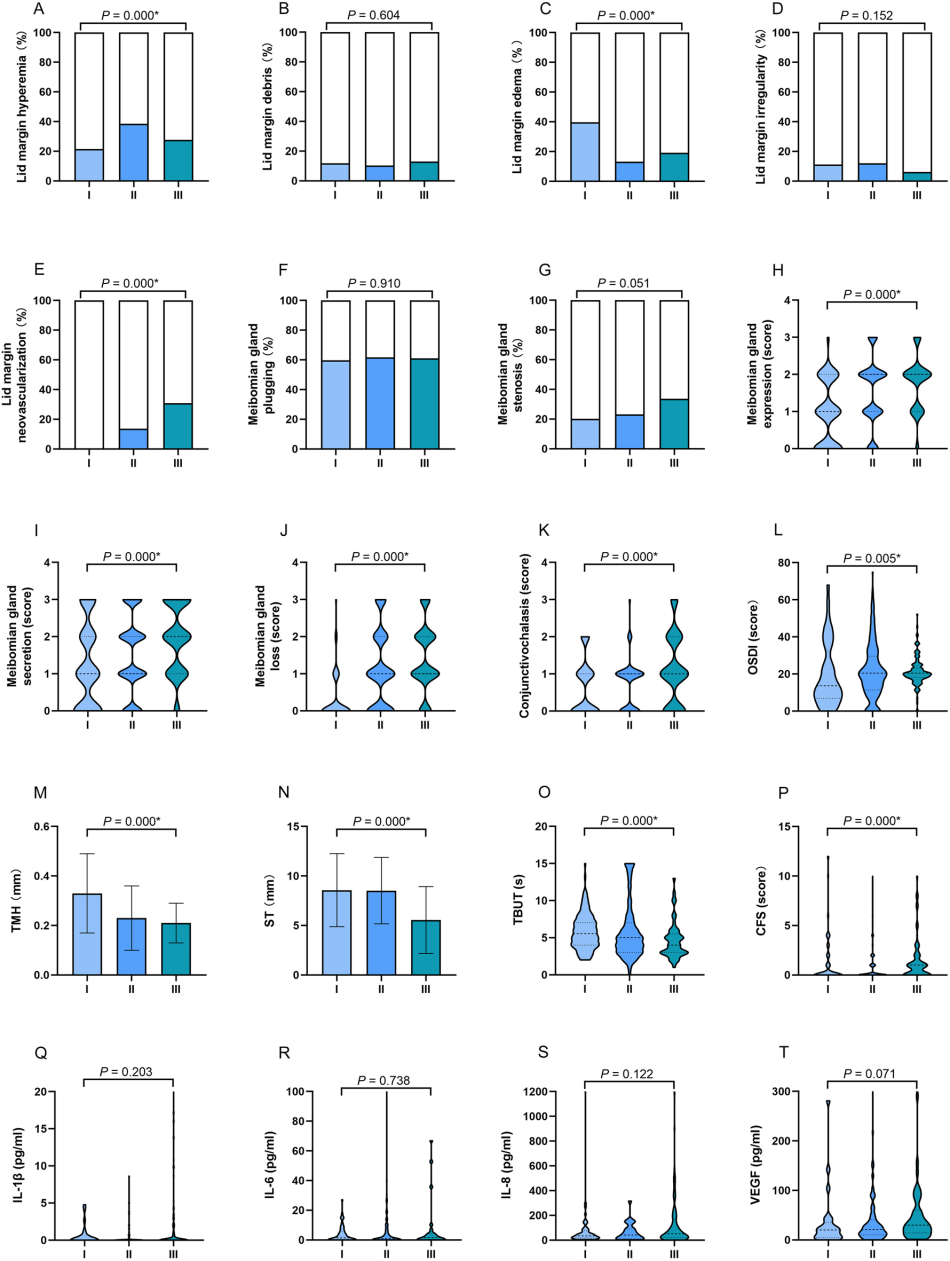
The ocular surface status and cytokine levels of the AQI level groups during long-term exposure. Individuals in Level II group exhibited a high proportion of lid hyperemia (**A**), Level I group exhibited a high proportion of eyelid edema (**C**), Level III group demonstrated a relatively high proportion of eyelid neovascularization (**E**), higher levels MG expression (**H**), MG secretion (**I**), MG loss (**J**), and conjunctivochalasis (**K**) than those in other groups. Individuals in Level III group had a significantly higher OSDI (**L**), lower TMH (**M**) and ST (**N**), shorter TBUT (**O**), and higher CFS score (**P**). The concentrations of IL-1β, IL-6, IL-8, and VEGF in three groups showed no difference. The results were expressed as percentage in A-G; as median with interquartile range in (**H**–**L**) and (**O**–**T**); as mean ± standard deviation (SD) in (**M**,**N**). AQI levels I: 0–50, II: 51–100, III: 101–150; *OSDI* ocular surface disease index, *TMH* tear meniscus height, *ST* Schirmer’s I test, *TBUT* tear film break-up time, *CFS* corneal fluorescein staining, *IL-1β* interleukin 1 beta, *IL-6* interleukin 6, *IL-8* interleukin 8, *VEGF* vascular endothelial growth factor, *P < 0.05.

Participants were divided into three groups according to the average AQI 1 month before the study, resulting in 144 individuals in level I, 574 in level II, and 146 in level III. The 1-month average AQI (AQI mean) of levels I, II, and III were 38.95 ± 8.86, 77.34 ± 10.59, and 108.99 ± 8.76, respectively. The ocular surface status and cytokine levels of the three groups, and the correlations between the ocular surface and AQI mean during long-term exposure are shown in Table 5 and Fig. 2.

**Table 5:** Correlations between the ocular surface status and air quality index (AQI) during long-term exposure.

	Level I	Level II	Level III	F	*P* Value	r	*P* Value
Average AQI (1-month)^$^	38.95 ± 8.86	77.34 ± 10.59	108.99 ± 8.76				
Numbers	144	574	146				
**Lid margin**							
Hyperemia/telangiectasia^#^	31 (21.5%)	221 (38.5%)	40 (27.6%)	17.87	0.000*	**0.108**	0.002*
Debris^#^	17 (11.8%)	59 (10.3%)	19 (13.0%)	1.01	0.604	0.031	0.366
Edema/thickening^#^	57 (39.6%)	75 (13.1%)	18 (19.2%)	53.62	0.000*	− **0.164**	0.006*
Irregularity^#^	16 (11.1%)	69 (12.0%)	9 (6.2%)	3.77	0.152	− 0.049	0.151
Neovascularization^#^	0 (0.0%)	78 (13.6%)	45 (30.8%)	57.64	0.000*	**0.402**	0.000*
**Meibomian gland**							
Plugging^#^	86 (59.7%)	354 (61.7%)	89 (61.0%)	0.19	0.910	0.132	0.221
Stenosis^#^	29 (20.1%)	133 (23.2%)	48 (33.6%)	7.76	0.051	0.066	0.055
**Meibomian gland expression**^**#**^							
0	53 (36.8%)	93 (16.2%)	7 (4.8%)	61.27	0.000*	**0.377**	0.000*
1	48 (33.3%)	170 (29.6%)	39 (26.7%)				
2	40 (27.8%)	239 (41.6%)	81 (55.5%)				
3	3 (2.1%)	72 (12.6%)	19 (13.0%)				
**Meibomian gland secretion**^**#**^							
0	48 (33.3%)	118 (20.6%)	9 (6.2%)	34.01	0.000*	**0.303**	0.000*
1	41 (28.5%)	177 (30.8%)	39 (26.7%)				
2	28 (19.4%)	163 (28.4%)	50 (34.2%)				
3	27 (18.8%)	116 (20.2%)	48 (32.9%)				
**Meibomian gland loss**^**#**^							
0	112 (77.8%)	192 (33.4%)	25 (17.1%)	108.07	0.000*	**0.404**	0.000*
1	24 (16.7%)	204 (35.6%)	59 (40.4%)				
2	6 (4.2%)	104 (18.1%)	43 (29.5%)				
3	2 (1.3%)	74 (12.9%)	19 (13.0%)				
Conjunctivochalasis^#^							
0	76 (52.8%)	218 (38.0%)	44 (30.1%)	26.03	0.000*	**0.163**	0.000*
1	47 (32.6%)	308 (53.6%)	57 (39.1%)				
2	21 (14.6%)	39 (6.8%)	32 (21.9%)				
3	0 (0.0%)	9 (1.6%)	13 (8.9%)				
OSDI (score)^$^	13.62 (6.82, 32.39)	20.45 (11.36, 29.55)	20.45 (18.18, 23.30)	10.58	0.005*	**0.125**	0.000*
TMH (mm)^$^	0.33 ± 0.16	0.23 ± 0.13	0.21 ± 0.08	67.09	0.000*	− **0.345**	0.000*
ST (mm)^$^	8.57 ± 3.69	8.52 ± 3.35	5.55 ± 3.38	89.08	0.000*	− **0.267**	0.000*
TBUT (s)^$^	5.84 ± 2.40	5.88 ± 3.74	4.65 ± 2.49	21.30	0.000*	− **0.126**	0.000*
CFS (score)^&^	0 (0, 1.5)	0 (0, 1)	1 (0, 2)	31.28	0.000*	**0.159**	0.014*
IL-1β (pg/ml)^&^	0 (0,0)	0 (0, 0.26)	0 (0, 1.18)	3.19	0.203	0.068	0.332
IL-6 (pg/ml)^&^	1.39 (0, 5.57)	0.88 (0, 5.62)	1.54 (0, 4.28)	0.61	0.738	− 0.003	0.965
IL-8 (pg/ml)^&^	34.99 (17.68, 77.41)	41.18 (11.56, 90. 44)	51.15 (20.04, 169.72)	4.21	0.122	0.038	0.589
VEGF (pg/ml)^&^	20.17 (4.41, 35.57)	20.89 (10.42, 40.66)	29.85 (14.34, 59.50)	5.29	0.071	**0.198**	0.004*

*OSDI* ocular surface disease index, *TBUT* tear film break-up time, *ST* Schirmer’s I test, *CFS* corneal fluorescein staining, *TMH* tear meniscus height, *IL-1β* interleukin 1 beta, *IL-6* interleukin 6, *IL-8* interleukin 8, *VEGF* vascular endothelial growth factor.

^#^Crosstab analyses were compared and results were expressed as number (percentage); ^$^Kruskal–Wallis tests were compared among three groups, independent *t* tests between two groups and results were shown as mean ± standard deviation (SD); ^&^Kruskal–Wallis tests were compared among three groups, Mann–Whitney nonparametric *U* tests between two groups and results were shown as median (25% quantile, 75% quantile); Spearman correlation analysis was conducted to assess the associations between different atmospheric environments, various indices of ocular surface, and cytokine levels.

*P < 0.05.

Individuals in Level II group exhibited a high proportion of lid hyperemia (II: 38.5%, III: 27.6%, I: 21.53%, *P* = 0.000). Individuals in Level I group exhibited a high proportion of eyelid edema (I: 39.6%, III: 19.2%, II: 13.1%, *P* = 0.000). Participants in Level III group demonstrated a relatively high proportion of eyelid neovascularization (III: 30.8%, II: 13.6%, I: 0%, *P* = 0.000). Regarding MG expression, MG secretion, MG loss, and conjunctivochalasis, individuals in level III group exhibited higher levels than those in other groups (all *P* = 0.000). Similar with eyelid and MG evaluation, individuals in Level III group had a significantly higher OSDI (median in III: 20.45, II:20.45, I: 13.62, *P* = 0.005), lower TMH (III: 0.21 ± 0.08, II: 0.23 ± 0.13, I: 0.33 ± 0.16, *P* = 0.000) and ST (III: 5.55 ± 3.38, II: 8.52 ± 3.35, I: 8.57 ± 3.69, *P* = 0.000), shorter TBUT (III: 4.65 ± 2.49, II: 5.88 ± 3.74, I: 5.84 ± 2.40, *P* = 0.000), and higher CFS score (median in III: 1, II: 0, I: 0, *P* = 0.000), Individuals in Level III group exhibited the highest concentrations of IL-1β, IL-6, IL-8, and VEGF. However, the difference was not significant.

Eyelid neovascularization (r = 0.402), MG expression (r = 0.377), MG secretion (r = 0.303), MG loss (r = 0.404), and TMH (r = − 0.345) were moderately correlated with AQI mean (*P* < 0.01).

## Discussion

In this study, MGD signs (eyelid neovascularization, MG plugging, MG expression, MG secretion, and MG loss) in the participants from oil region were more frequent and severe compared with other regions. At the same time, individuals in oil region showed a significant reduction in the TMH, ST, and TBUT, and a significant increase in CFS, IL-1β, IL-6, IL-8, and VEGF concentration. In the long term, individuals exposed to higher AQI levels presented with MGD more commonly.

At present, many studies have reported that DED is related to air pollution^28^. As Mo et al. reported, PM_2.5_, PM_10_, SO_2_, NO_2_, and CO are strongly related with DED, while O_3_ was not^18^. However, Hwang et al. suggests that higher O_3_ and lower humidity were associated with DED in Korean, while PM_10_ was not^29^. NO_2_ and PM_2.5_ were also found to cause tear film stability and osmolality reduction^30^. Another study by Um et al. found that only SO_2_ was associated with DED in South Korea, while NO_2_, O_3_, CO, and PM_10_ were not^31^. Nevertheless, the impact of air pollutants on ocular surface remains controversial. In our study, lower temperature, higher O_3_, and higher SO_2_ levels in oil region are theorized to be contributing factors. Our result suggested that reduced temperatures might have favored the lower TBUT, more serious lid margin neovascularization, MG plugging and MG secretion. A possible reasonable explanation is that low temperatures may cool down the MG orifice surface, which results in the transient blockages of orifices, premature condensation of secretions and an increase in the viscosity after being discharged from MG orifices, leading to tear film instability and serious MGD signs^32^. However, the more serious lid margin hyperemia, debris and edema were seen in a relatively higher temperatures, suggested that elevated temperatures might favor the lid margin abnormality and inflammation. The results seemed to be a paradox. A human study was similar with our results that showing increased lipid layer thickness and TBUT but a high tear evaporation rate at a higher temperature^32^. Therefore, appropriate temperature is recommended for ocular surface health. O_3_ possesses the property of powerful oxidation and could affect the eye, skin, and respiratory system. Even at low concentrations, exposure to environmental O_3_ directly can be hazardous. A cross-sectional study from Korea suggested that higher O_3_ levels were strongly related to DED^29^. According to Lee et al., long-term exposure to a high concentration of O_3_ induces oxidative damage, enhances inflammatory cytokine levels in tears, and decreases tear production and conjunctival goblet cell density, known to be via the NF-κB pathway in vitro^33,34^. Additionally, in SOD1 (−/−) mice, increased periglandular inflammatory infiltrates, increased fibrosis, decreased glandular acinar density, and apoptosis were found in MGs^35^. Those previous researches in agreement with our results, the increased O_3_ concentration might contribute to the elevated cytokine levels (IL-1β, IL-6, IL-8), decreased ST, TMH and TBUT, higher OSDI and CFS scores, and serious MGD signs (MG loss and margin vascularization). Ozone is such a small molecule that it may approach the ocular surface and lacrimal glands, then induce inflammation and tear secretion abnormality. According to the studies of Wolkoff et al., aggressive aerosols and combustion products, such as SO_2_, could alter the structural composition of the outermost lipid layer of the precorneal tear film and cause eye burning, drying, and itching, resulting in DED in most cases^15^. The results of this study demonstrated SO_2_ concentrations were associated with more serious MGD (lid vascularization), lower TBUT, higher OSDI and CFS scores, and higher VEGF concentration, which is in accordance with the previously reported findings. Therefore, it is speculated that lower temperature induces temporary blockages of orifices, SO_2_ alters the lipid layer of the tear film, and excessive oxidative stress exacerbates inflammation and the chance of microbial infection.

In this study, individuals in oil regions presented more frequent and severe MGD, along with an increased level of cytokines, including IL-1β, IL-6, IL-8, and VEGF. Cytokine fluctuations were significant associated with air pollutants, such as PM, O_3_, and SO_2_ levels. The fluctuations in the cytokine concentrations showed a strong correlation with ocular surface changes, including conjunctivochalasis, TBUT and CFS, among others. These indicated that the expression of proinflammatory cytokines was sensitive to air pollutant changes, especially gases. Solomon et al. have detected an increase in the proinflammatory forms of IL-1 (IL-1α and mature IL-1β) in the tear fluid of patients with DED, suggesting that IL-1 may play a key role in the pathogenesis of keratoconjunctivitis sicca^36^. Barton et al. demonstrated an increased expression of IL-1, IL-6, and IL-8, and decreased epidermal growth factor levels in eyes with Sjögren's syndrome^37^. Lee et al. demonstrated that the expression of IL-6, IL-8, IL-17, and IFN-γ increased after exposure to O_3_ in cultured human conjunctival epithelial cells not pretreated with IL-1α^34^. Generally, IL-1β, IL-6, and IL-8 are considered to induce an oxidative stress response^34^. IL-1 and IL-6 jointly promote Th-17 cell differentiation, and IL-6 is also associated with the B-cell activation, proliferation, and differentiation^34^. The chronic inflammation is associated with high tear cytokines concentrations^38–40^, and both of them are important in the pathogenesis of MGD^41^. Therefore, the tear cytokines may play key roles in the pathogenesis of MGD exposed on air pollution.

According to the WHO, PM concentration in China have reached “bad” or even “very bad” levels, particularly in those industrial and densely populated areas. PM is identified as a crucial indicator of environment pollution and should draw public attention^19,42^. Previous studies demonstrated that high levels of PM_2.5_ and PM_10_ were associated with decreased TBUT and conjunctival goblet cells density and increased pro-inflammatory cytokines in mice^30,43^, which were in accordance with our results. There were some correlations between PM concentration fluctuations and MG morphology/function and tear quantity/stability. These results suggested that PM_2.5_ and PM_10_ were positively related to the severity of ocular surface pathologies and MGD, we suspect that the PM may first affect the MG, leading to abnormal morphology and secretory function, thus affecting the tear film stability and ocular surface, resulting in ocular surface discomfort. Fu et al. reported that PM_2.5_ has a time- and dose-dependent effect on cytotoxicity in cultured human corneal epithelial cells^44^. Thus, it may be difficult to detect ocular surface differences when the short-term changes in PM are not obvious. Yoon et al. have found that the effects of collected road dust on cellular responses are strongly dependent on their concentration and solubility^45^, indicating that atmospheric PM concentrations cannot be equal to PM concentrations in tears. Rather than PM concentrations, the components of PM are more important^45,46^. The specific compositions of PM, such as polyaromatic hydrocarbons, elemental/organic carbon, heavy metals, nitrate and sulfate^47^, change according to different zones and time. And the chemical characteristics of the compounds adsorbed to the particle surface will definite affect the PM toxic effects. In our study, the concentrations of PM_2.5_ and PM_10_ in the oil region were higher than those in the living and steel regions but lower than those in the coal region. However, the ocular surface presented more serious signs, and cytokine concentrations are higher than the three regions. We suspect that the air pollutants in the oil region are more soluble and complex, especially when affected by weather conditions, atmospheric chemistry, and complicated interactions with multiple air pollutants (such as O_3_ and SO_2_)^47^.

This study attempts to reflect the long-term pollution status by using the average preliminary AQI. Previous studies reported dose–response relationships in the constant concentration of air pollutants^48,49^. However, the exposure on the ocular surface is continuous, and we used the mean AQI in the present study inevitably. Symptoms and signs of MGD gradually increased with the 1-month average AQI, especially the lid margin neovascularization, MG expression, MG secretion, and MG loss. A certain transition period between the onset of DED and MGD signs is suspected, and long-term air quality likely plays an important role in the process. However, the AQI reflects the pollution levels of the most influential pollutants daily, which the 1-month AQI may not have captured.

The study had several limitations. First, the study's sample size was not large enough, making it difficult to stratify the differences in temperature and relative humidity for further analysis. Second, the study was a prospective cohort study, so the results did not definitively provide causal evidence between MGD and air pollutants. Third, the air pollutants exposure on the ocular surface is continuous. However, it is hard to monitor air quality personally and constantly in a large amount of people. We used the daily basis in the present study inevitably. There were differences between the indoor and outdoor activities of individual. However, our participants were asked to do 3–4 h outdoor activities to eliminate this difference. Despite some limitations, we believe this study is meaningful because it is a well-designed multicenter prospective study with organized statistical analysis. We investigate different air pollutants, ocular surface parameters and inflammation cytokines in different areas across China, and found it necessary to make a protocol to handle those air pollutants as much as possible to decrease their harmful effects.

In conclusion, individuals in the oil region presented more frequent and severe MGD, along with higher cytokine levels. MGD is likely in long-term exposure to relatively high AQI. The significant association between air pollution and ocular surface discomfort indicates the urgency to accelerate the environmental protection.

## Data Availability

The datasets used and/or analyzed during the current study are available from the corresponding author on reasonable request.
